# Stem cell therapy for vocal fold regeneration after scarring: a review of experimental approaches

**DOI:** 10.1186/s13287-022-02853-9

**Published:** 2022-05-03

**Authors:** Mikhail V. Svistushkin, Svetlana Kotova, Anastasia Shpichka, Svetlana Starostina, Anatoliy Shekhter, Polina Bikmulina, Anna Nikiforova, Anna Zolotova, Valery Royuk, P. A. Kochetkov, Serge Timashev, Victor Fomin, Massoud Vosough, Valery Svistushkin, Peter Timashev

**Affiliations:** 1 Department for ENT Diseases, Sechenov University, Moscow, Russia; 2 World-Class Research Center “Digital Biodesign and Personalized Healthcare”, Sechenov University, Moscow, Russia; 3 Institute for Regenerative Medicine, Sechenov University, Moscow, Russia; 4 Department of Polymers and Composites, N.N. Semenov Federal Research Center for Chemical Physics, Russian Academy of Sciences, Moscow, Russia; 5 Chemistry Department, Lomonosov Moscow State University, Moscow, Russia; 6 University Hospital No 1, Sechenov University, Moscow, Russia; 7 National Research Nuclear University «MEPhI», Moscow, Russia; 8 Department of Internal Medicine No 1, Sechenov University, Moscow, Russia; 9 Department of Regenerative Medicine, Cell Science Research Center, Royan Institute for Stem Cell Biology and Technology, ACECR, Tehran, Iran

**Keywords:** Vocal folds, Cell therapy, Scarring, Mesenchymal stromal cells, Experimental studies

## Abstract

This review aims at becoming a guide which will help to plan the experimental design and to choose adequate methods to assess the outcomes when testing cell-based products in the treatment of the damaged vocal folds. The requirements to preclinical trials of cell-based products remain rather hazy and dictated by the country regulations. Most parameters like the way the cells are administered, selection of the cell source, selection of a carrier, and design of in vivo studies are decided upon by each research team and may differ essentially between studies. The review covers the methodological aspects of preclinical studies such as experimental models, characterization of cell products, assessment of the study outcome using molecular, morphological and immunohistochemical analyses, as well as measuring the tissue physical properties. The unified recommendations to perform preclinical trials could significantly facilitate the translation of cell-based products into the clinical practice.

**Graphical abstract:**

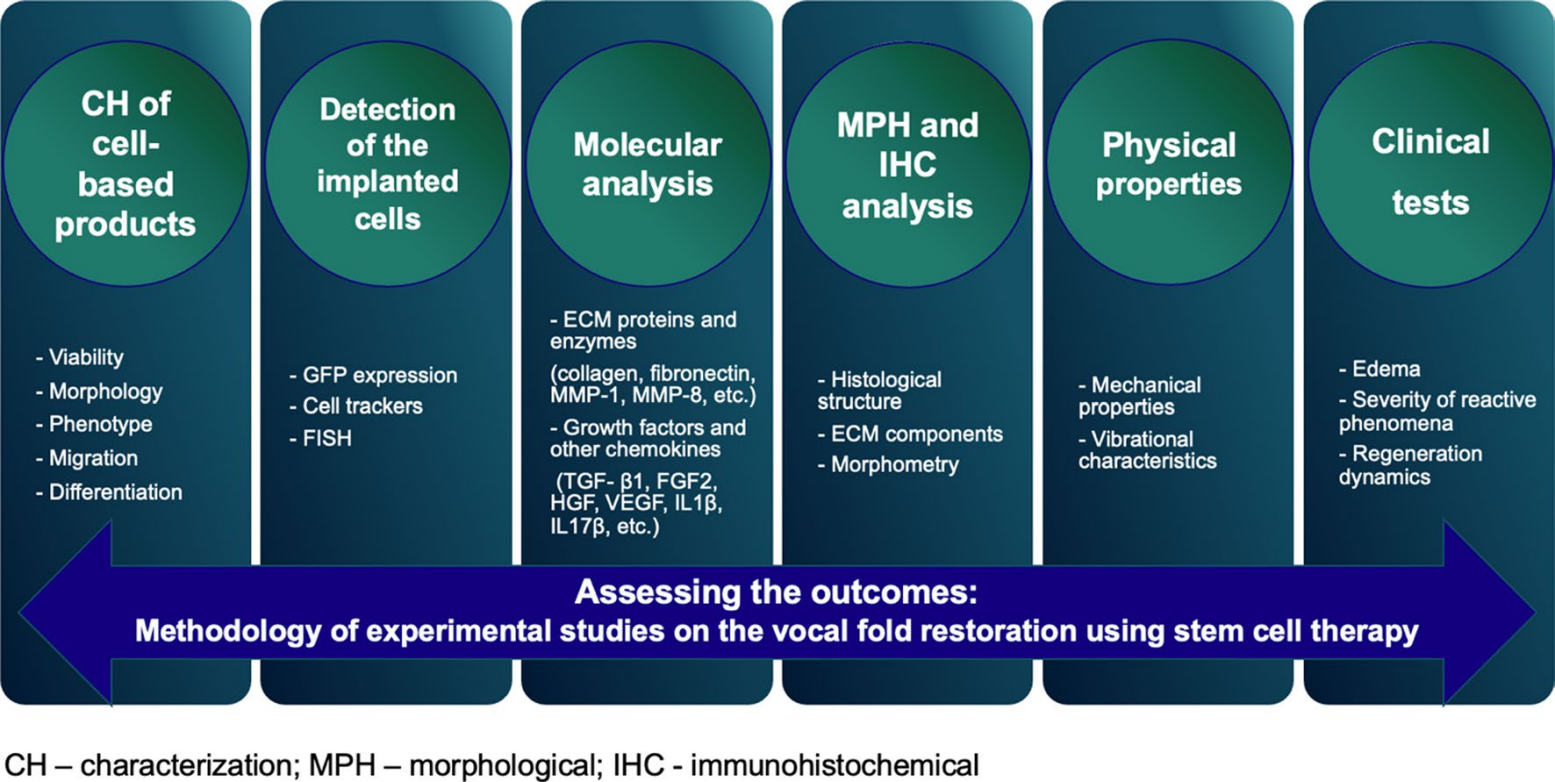

## Introduction

**Fig. 1:**
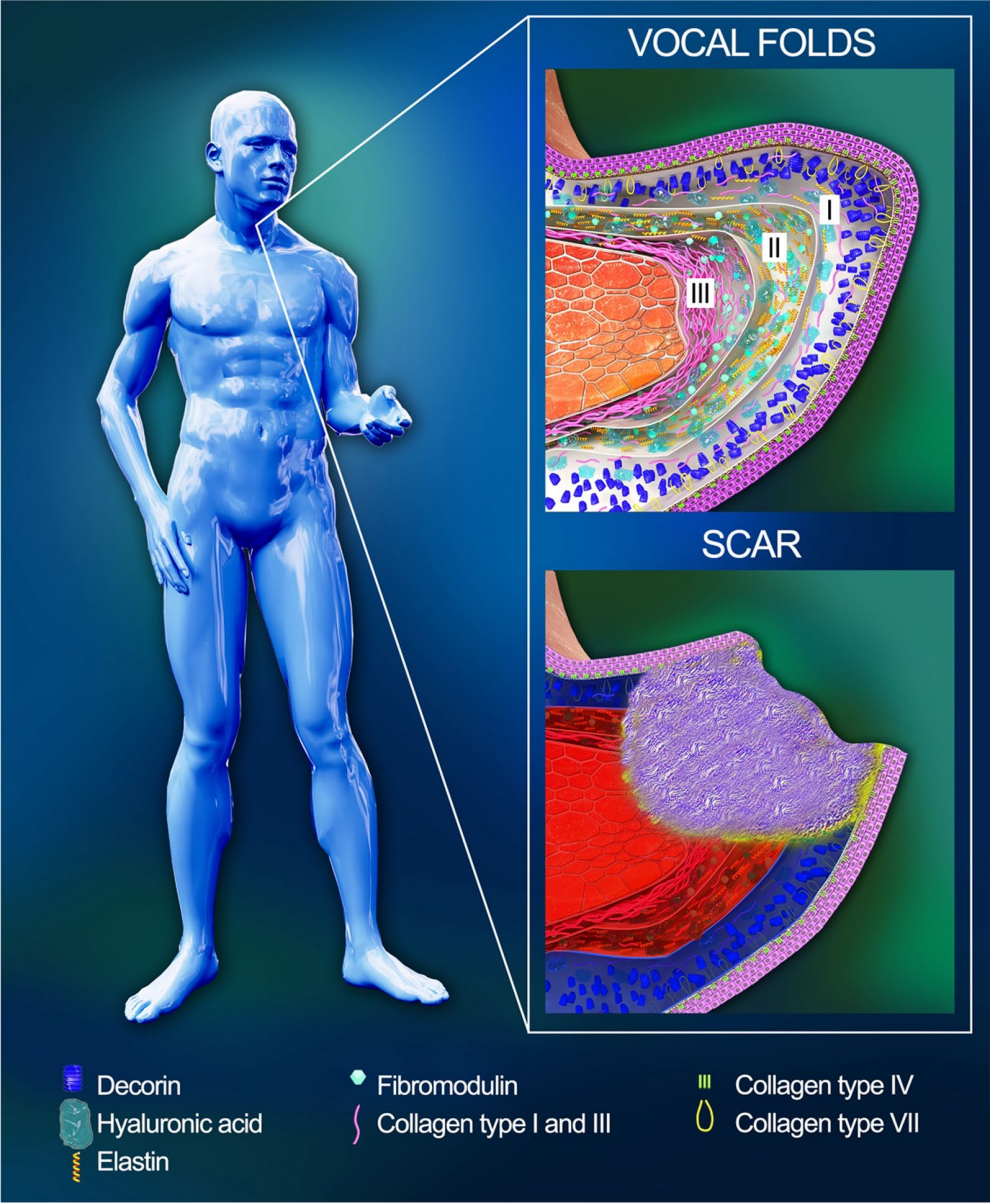
Structure of the normal and damaged vocal folds. The VF are covered with the squamous epithelium separated by the basement membrane from the lamina propria and muscle vocalis. The lamina propria consists of three layers: superficial (Reinke’s space) (I), intermediate (II), and deep (III)

Scarring and atrophy of the vocal folds (VF) is a wide-spread and simultaneously one of the most complex problems in otolaryngology. It may lead to prolonged and frequently irreversible impairment of the vocal function. The causes are extremely diverse and include acute and chronic inflammation, voice overuse, trauma of any etiology, endotracheal intubation, presbyphonia, etc. [1–3]. VF scars are characterized by spatial disorganization and a quantitative disbalance of the extracellular matrix proteins in the lamina propria of the mucosa, which are replaced predominantly with thickened and chaotically distributed bundles of collagen type I (Fig. 1). This leads to higher rigidity and density of the tissue [2, 4]. In turn, the VF lose their unique rheological characteristics needed for the generation of mucosal waves and production of sounds, respectively [5–9].

As of now, a number of techniques have been developed which allow partial restoration of these properties and, correspondingly, the acoustical characteristics of the voice in patients with VF scarring. These techniques may be divided into two categories: conservative (phonopedics and pharmaceutical treatment) and surgical interventions (phonosurgery). However, in spite of the existing variety of the treatment approaches, the functional result of the VF scarring therapy is unpredictable and limited, since none of the known approaches results in the restoration of the native structure of the mucosa’s lamina propria [2, 5, 6, 9].

New technologies belonging to regenerative medicine aim at solving the problem of restoration of the morphofunctional properties of the damaged tissue based on the application of stem cells [10, 11].

The potential of the cell therapy of such lesions is based on a number of prerequisites related to the properties of the used cells, specifics of the VF structure, as well as the pathophysiological processes during scarring. The lamina propria of the VF is sharply divided into three layers. Those layers are distinct in their qualitative and quantitative composition and the 3D arrangement of the extracellular matrix (ECM) [12–16]. Significant changes proceed after the VF injury [17, 18], and the observed processes in the first several days after the injury reflect a peculiar critical point which determines the subsequent course of repair and the final functional result. This fact dictates the necessity of a search and development of approaches to prevent scarring at early stages [18–21]. In turn, these processes represent a logical chain of reactions in which anti-inflammatory cytokines being initially released from thrombocytes provide the chemotaxis of neutrophils and macrophages that leads to the activation of fibroblasts synthesizing the ECM components [19]. The stem cells’ ability to modulate the course of the inflammatory response and the profile of the synthesized ECM components in the early times after the injury underlies the interest to their application in the therapy of the VF lesions. It should be noted that a great attention has been paid recently to the paracrine mechanisms of their action, since their viability after implantation to the VF is generally rather low [22–27]. For instance, a study by Hiwatashi et al. (2017) showed that the medium in which human bone marrow-derived MSC were cultured inhibited the profibrotic effects of fibroblasts in the VF stimulated by TGF-β1; this action was realized via the change in the activity of TGF-β linked cell signals (including Smad signaling pathways) [28].

For the last several years, the efficiency of stem cells in the treatment of VF scarring has been demonstrated in a large number of experimental studies [29–32]. However, the number of conducted clinical trials remains somewhat small [33–35], which may be related to the essential differences in the experimental design and, in general, to the lack of a standard approach to the estimation of the results of the VF repair. In particular, a wide variety of animals (rabbits, dogs, rats, etc.) are used in experimental models, and application of immunosuppressants is allowed [22, 36–41]. Then, the results are analyzed using different sets of methods. Some researchers restrict themselves to histological studies only, while others additionally estimate gene expression and mechanical and vibrational characteristics of the repaired tissue. While in clinical practice the voice restoration is assessed by the acoustic analysis, special questionnaires (e.g., VHI), videostroboscopy, the animal experiments lack any definitive recommendations [23, 25, 38, 41–44].

This review focuses on the methodological component of the experimental studies on the VF restoration using stem cell therapy, as well as on the recommendations for adequate assessment of their results. The presented analysis of the published data may facilitate planning the experimental studies on the treatment strategies for VF scarring.

## Overview of experimental studies

**Fig. 2:**
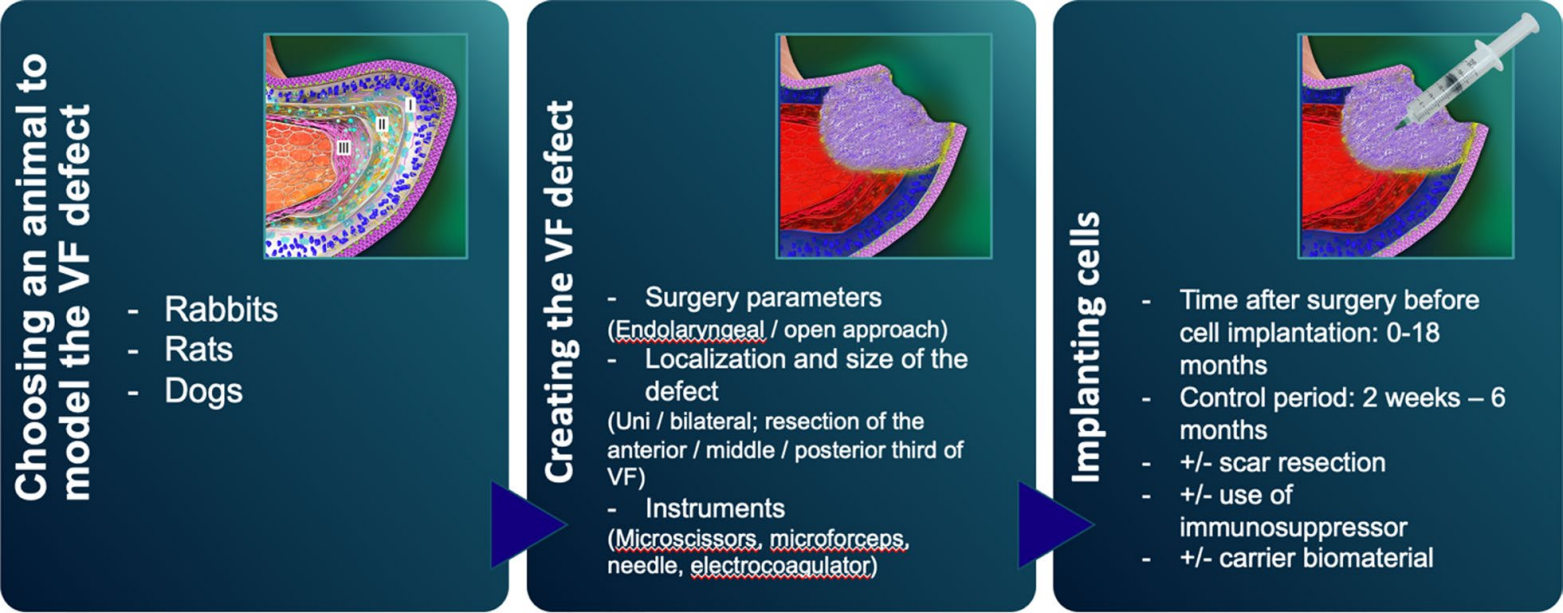
Design of experimental studies aiming to reveal the efficacy of stem cell therapy in the VF scarring. The study usually includes three main steps: choice of an animal model, injury the VF for the scar formation, and cells’ implantation

The published experimental studies (Fig. 2, Table 1) are dedicated to finding the specifics of the VF tissue regeneration in the presence of stem cells. Therefore, the common feature of these studies is infliction of a VF injury by one or another technique.

**Table 1:** Experimental models used to study cell-based products in the treatment of VF scarring

	Animals (*n*)	Surgery type	Time after surgery before IMP, days	Cell type/carrier	Dose	Duration	Use of IS
Kanemaru et al. [49]	Rats (4)	Exterior access Injury with a 32G needle	0	Xenogeneic (murine) BM-MSC expressing GFP	(1–3) × 10^5^	8 weeks	No
Hertegard et al. [23]	Rabbits (10)	Endolaryngeal Limited unilateral resection of VF with microforceps and microscissors	0	Xenogeneic (human) BM-MSC	0.8 × 10^5^ cells in 0.1 mL	4 weeks	Yes, Tacrolimus
Lee et al. [45]	Dogs (10)	Endolaryngeal Bilateral resection of the posterior third of VF with an electrocoagulator	4	Autologous AD-MSC/atelocollagen	(1.0–3.0) × 10^6^ cells in 0.2 mL	8–24 weeks	No
Johnson et al. [52]	Nude rats (12)	Endolaryngeal Bilateral injury of the middle third of VF with a 27G needle	30	Xenogeneic (murine) BM-MSC expressing GFP/HA-based sECM	ND	4 weeks	No
Xu et al. [46]	Rabbits (40)	Endolaryngeal Limited resection of the anterior and middle third of VF	3–5	Autologous AD-MSC/collagen or HA	ND	15, 40 days and 3, 6, and 12 months	No
Svensson et al. [37]	Rabbits (12)	Endolaryngeal Limited bilateral VF resection using microforceps	63	Xenogeneic (human) BM-MSC	(0.8–1.0) ˣ 10^5^ cells in 0.1 mL	10 weeks	Yes, Tacrolimus
Ohno et al. [50]	Dogs (12)	Endolaryngeal Bilateral VF resection using microforceps and microscissors	60	Autologous AD-MSC/atelocollagen sponge	1 × 10^6^ cells in 5 × 3 × 3-mm	6 months	No
Kim et al. [60]	Rabbits (24)	Endolaryngeal Local uni/ bilateral VF resection using microinstruments	0	Xenogeneic (murine) BM-MSC expressing GFP	1 × 10^5^ cells in 50 μL	3 months	No
Kim et al. [38]	Rabbits (40)	Endolaryngeal Local uni/ bilateral VF resection using microinstruments	0	Xenogeneic (human) AD-MSC/Alginate-HA hydrogel	1 × 10^6^ cells in 50 μL	3 months	No
Peng et al. [27]	Dogs (14)	Endolaryngeal Bilateral resection of the middle third of VF using a laser	0	Allogeneic MSC from epiglottis mucosa/collagen	2 × 10^6^ cells in 0.2 ml	2–8 weeks	No
Choi et al. [25]	Rabbits (24)	Endolaryngeal Bilateral VF injury with an electrocoagulator	0	Xenogeneic BM-MSC/Small intestine submucosa	2 × 10^7^ cells in 50 μL	8 weeks	No
Hu et al. [39]	Dogs (17)	Endolaryngeal Limited resection of the anterior and middle thirds of VF	ND	Autologous AD-MSC, vocal fold fibroblasts, differentiated AD-MSC	(3.0–4.0) × 10^5^ cells in 3–4 ml	15 and 40 days and 3 and 6 months	No
Hiwatashi et al. [40]	Rats (70)	Endolaryngeal Limited unilateral VF resection using microscissors	60	Allogeneic AD-MSC and BM-MSC	5.0 × 10^5^ cells in 50 μL	1 and 3 months	No
Svensson et al. [57]	Rabbits (16)	Endolaryngeal Limited unilateral VF resection using microforceps	0	Xenogeneic (human) ESC	10^4^ cells in 0.1 mL	6 weeks, 3 months	Yes, Tacrolimus
Valerie et al. [53]	Rabbits (74)	Endolaryngeal Limited bilateral resection of the anterior and middle thirds of VF using microforceps	540 (18 months)	Autologous AD-MSC	1.0 × 10^4^ cells in 0.1 mL	3 months	No
De Bonnecaze et al. [41]	Rabbits (20)	Exterior (median thyroidotomy) Unilateral injury of left VF with an electrocoagulator	0	Autologous AD-MSC	2 × 10^6^ cells in 0.1 mL	6 weeks	No
Shiba et al. [47]	Rabbits (8)	Exterior (midline laryngofissure) Unilateral removal of the mucosa along the whole VF length	0	Autologous AD-MSC/fibrin gel	NA	4 weeks	No
Imaizumi et al. [26]	Rats (30)	Endolaryngeal Unilateral VF injury with a 25G needle	0	Xenogeneic human iPSC/HA hydrogel	1.0 × 10^5^ cells in 5 μL	1 and 2 weeks	No
Morisaki et al. [61]	Rats (72)	Endolaryngeal Unilateral VF injury with a 25G needle	0	Allogeneic AD-MSC	5.0 × 10^5^ cells in 50 μL	3, 14, 56 days	No
Bartlett et al. [54]	Rabbits (84)	Endolaryngeal Bilateral resection of the middle VF third using microforceps	42	Xenogeneic (human) BM-MSC/HyStem-VF hydrogel	3 × 10^5^ cells in 100 μL twice or 1.5 × 10^5^ cells in 100 μL	2, 4, and 10 weeks	No
Goel et al. [48]	Rabbits (8)	Exterior (midline laryngofissure) Unilateral removal of the mucosa along the whole VF length	0	Allogeneic AD-MSC/fibrin gel	NA	4 weeks	No
Svistushkin et al. [62]	Rabbits (12)	Endolaryngeal Limited unilateral VF resection using microforceps	84	Autologous BM-MSC	6 × 10^5^ cells in 0.6 mL	12 weeks	No
Hertegård et al. [22]	Rabbits (18)	Endolaryngeal Limited VF resection using microforceps	0	Xenogeneic (human) BM-MSC/HA hydrogel	1 × 10^5^ cells in 0.1 mL	3 and 25 days	No

*AD-MSC* adipose tissue-derived mesenchymal stromal cells, *BM-MSC* bone marrow-derived mesenchymal stromal cells, *ESC* embryonic stem cells, *GFP* green fluorescent protein, *HA* hyaluronic acid, *IMP* implantation, *iPSC* induced pluripotent stem cells, *IS* immunosuppressor, *sECM* synthetic extracellular matrix, *VF* vocal folds

The choice of the laboratory animal and experimental model of the VF injury represents one of the key points in the design of a preclinical study. In relevant studies, the following animals are used (in the order of decreasing frequency of application): rabbits, rats, and dogs [29, 37, 45, 46]. A typical experimental model involves the VF tissue injury with the subsequent implantation of the cell material either with or without a carrier [29, 31]. In the majority of studies, the VF defect is created with a cold microinstrument (forceps or scissors); in some cases, a surgical coagulator or a laser is used. The injury of the VF mucosa is inflicted in the region of the anterior and middle third of the VF and includes the superficial layers of the thyroarytenoid muscle; however, in a number of studies, e.g., in the works by Shiba et al. (2016) and Goel et al. (2018), the mucosa is removed along the whole VF length [29, 31, 47, 48].

The implant administration is performed via an injection; as a rule, the endolaryngeal access is used, rarely—an exterior access [41, 49]. When using hydrogel-based systems or tissue engineered constructs, they are implanted in a subepithelial pocket of the VF via the endolaryngeal route, or fixed in the region of the resected VF after a median thyrotomy [24, 33, 48, 50]. In the prevailing number of studies, the product is administered to the acute primary wound of the VF immediately post-injury. In some studies, the implantation is performed in early times—during the first week, or later—in 1–2 months. Rarely, other times are seen, such as in 18 months, or 4 days before the injury [24, 37, 39, 40, 45, 46, 50–54].

In a review study by Mattei et al. (2017), the authors note that a two-stage design of the experiments, i.e., the delayed implant administration, is more consistent with the conditions of the clinical practice, since the treatment of scars should not be started earlier than in 6 months [29]. In the mentioned research works, the cell product was administered not earlier than in 2 months after the defect creation, i.e., during the phase of scar tissue remodeling. Only in one study (Svensson et al. 2011), a preliminary excision of the scar tissue was performed [37]. In all the other cases, the implantation was performed directly into the VF scarification site via an injection of a cell suspension in a buffer solution or in a carrier (e.g., hyaluronic acid based), which may influence the volume and viscoelastic properties of tissues [24, 37, 40, 52, 53]. These peculiarities should be taken into account, since, in the case of utilizing cells without a carrier, the therapy effects may be explained only by the cells’ influence on the ECM remodeling in the already relatively mature scar tissue that is of great importance when extrapolating the results to the clinical studies. The latter must minimize the potential risks of aggravating fibrosis by the surgical injury.

The studies on the VF regeneration are presented in the literature based on the type of a cell product, from the viewpoint of the cell source in respect to the recipient—laboratory animal. In most studies, xenogeneic stromal or stem human cells are used: mesenchymal stromal cells (MSC) derived from the bone marrow or adipose tissue, embryonic stem cells, induced pluripotent stem cells. Such an approach is in agreement with the position of the European Medical Agency (EMA), established in the analytical document with the requirements on the quality of preclinical and clinical trials of medical products based on stem cells (EMA/CAT/571134/2009). The document states that, in order to prove a concept, the most suitable approach would be the use of human cells, since it is this product that will be potentially applied in the clinical practice [22, 23, 26, 38, 54–57].

At the same time, application of a xenogeneic cell material in animals of non-immunodeficient lines may potentially affect the final result. Nevertheless, the immunosuppressive therapy (with the use of tacrolimus) was utilized only in one series of experimental studies; according to the authors’ data, the use of tacrolimus may reduce the antifibrotic effects of human MSC upon their implantation into the damaged VF of a rabbit [23, 37, 55, 58]. Kim et al. in their article point to the absence of signs of the inflammatory reaction upon implantation of adipose tissue-derived human MSC in a gel based on hyaluronic acid and alginate into the rabbit VF and explain the observed effect by the immunomodulating properties of MSC, referring to the study by Ryan et al. [38, 59].

In a number of publications, the researchers use MSC derived from the bone marrow of “green” transgenic mice whose cells express green fluorescent protein, which makes it possible to detect the cell material in specimens by means of fluorescent microscopy. Nevertheless, such cells are xenogeneic in respect to other species. In this connection, Kanemaru et al. and Johnson et al. applied immunodeficient lines of rats, and the corresponding immunosuppressive therapy was not used [49, 52].

Besides, to repair the VF damage, MSC derived from the adipose tissue, bone marrow and mucosa of the epiglottis were applied, as well as differentiated fibroblast-like cells from the adipose tissue [27, 39, 41, 45, 46, 50, 53].

It should be noted that the number of implanted cells differs drastically in different studies and is found in the range of 1 × 10^4^ to 2 × 10^7^ cells. The most frequently mentioned dose is 1 × 10^5^ дo 1 × 10^6^. Only in one study, by Bartlett et al., a comparison of two different doses of human bone marrow-derived MSC was performed, 1.5 × 10^5^ and 3 × 10^5^ cells [25, 53, 54]. The estimation of the results in the majority of studies was performed 1–3 months post-implantation [36, 40, 45, 46, 51, 53, 54].

## Characterization of cell-based products before implantation

To characterize the used cell products and confirm the cells’ phenotype (Table 2), many authors apply flow cytometry. The following panel of markers is used most frequently: CD34, CD45, CD73, CD90, CD105. At the same time, only few authors present their numerical results with a statistical data processing [27, 61], although it is important for the confirmation of the analysis objectivity. Besides, it would be reasonable to expand this panel with the following markers (entirely or partially): CD29, CD166, CD44, HLA-I, Sca-1, CD14, CD11b, CD19, CD31, CD80, CD106, and HLA-II. Such a difference hinders the comparative analysis; moreover, the cases of contradictory results on the expression of one and the same marker are observed, in particular, CD34 in MSC from the rabbits’ adipose tissue [41, 46].

**Table 2:** A methodological pattern of studies on assessing the effects of cell-based products’ implantation into a defect of the vocal folds

	In vitro characterization	Cell type and detection method after IMP	Markers for MA	Histological staining	Markers for IHC	Morphometric parameters	In vivo assessment	Physical properties	
								Mechanics	Vibration
Kanemaru et al. [49]	FACS (Sca-l, CD29, CD34, CD44, CD45, CD4ge, and 7-AAD)	Xeno (murine) BM-MSC GFP tracing	No	H&E	Keratin Desmin	No	No	No	No
Hertegard, et al. [23]	FACS (CD73, CD90, CD105, CD166, CD14, CD31, CD34, CD45, and CD80)	Xeno (human) BM-MSC FISH	No	H&E	Collagen type I	Collagen density	No	No	No
Lee et al. [45]	No	Auto AD-MSC CM-Dil tracking	No	H&E	No	No	Direct laryngoscopy	No	No
Johnson et al. 2010 [52]	– FACS (CD11b, CD19, CD-45, 7-AAD) – AlamarBlue assay – ICC (CD-44, Sca-1)	Xeno (murine) BM-MSC GFP tracing	COL3A1 FN1 HAS3 HYAL3 SMA TGF- β1	No	No	No	No	No	No
Xu et al. [46]	– Phase contrast microscopy – MTT assay – FACS (CD34, CD44, CD105, CD106) – ICC (Vimentin) – HS (Oil red O, Von Kossa) – SEM	Auto AD-MSC DAPI tracking	No	H&E MT AB	FN	Collagen, HA, FN density	Endoscopy	No	No
Ohno et al. [50]	No	Auto BM-MSC ND	No	vG AB	No	LP thickness Collagen and HA density	No	No	No
Kim et al. [60]	No	Xeno (murine) BM-MSC GFP tracing	COL1A1 HAS2 FN1 TGF- β1	H&E MT AB	Collagen type I	Collagen and GAG density	Endoscopy	No	No
Kim et al. [38]	– FACS (CD73, CD90, CD31, CD34, CD-45) – Differentiation test (AG, OG, CG)	Xeno (human) AD-MSC CM-DiI tracking	No	H&E MT VvG	HGF FSP-1 Collagen type I FN	Collagen, elastin, and FN density	Endoscopy	No	No
Peng et al. [27]	– FACS (CD29, CD44, CD90, CD105e, CD34, CD45) – Clonogenicity assay – Differentiation test (AG, OG, CG)	Allo EM-MSC Dil tracking IHC (SMA, vimentin)	No	H&E MT vG AB	FN	Relative content of collagen, elastin and HA	Comparativeendoscopy	No	No
Choi et al. [25]	No	Xeno BM-MSC In vivo DiD tracking	–	H&E MT AB PR		Scar index measurement Intensity of collagen optical density	Endoscopy	No	No
Hu et al. [39]	– Phase contrast microscopy – ICC (vimentin, collagen)	Auto AD-MSC/VF fibroblasts/dAD-MSC ND	No	No	Collagen Elastin HA Decorin FN	No	Endoscopy	No	No
Hiwatashi et al. [40]	No	Allo (murine) BM-MSC and AD-MSC GFP tracing	FGF2 HGF COL1A1 COL3A1 HAS1 HAS2 HAS2 MMP1 VEGFa	vG AB	No	LP thickness collagen and HA density	No	No	No
Svensson et al. [57]	No	Xeno (human) ESC FISH	No	H&E	Collagen type I	LP thickness Collagen density Scored fibrosis analysis	No	No	No
Valerie et al. [53]	No	Allo AD-MSC ND	No	H&E MT AB RO	No	LP thickness Scored collagen, elastin and HA density	No	No	No
De Bonnecaze et al. [41]	– FACS (CD34, CD45, CD31, CD73, CD90) – Differentiation test (AG, OG)	Auto AD-MSC GFP tracing via LV transduction	No	H&E PR O	Collagen type I FN	LP and EP thickness PR optical density Density of elastic fibers Number of inflammation foci (foci/mm3)	No	No	No
Shiba et al. [47]	– HA (H&E, vG, MT) – IHC (vimentin, panCK)	Auto AD-MSC in fibrin gel ND	No	H&E vG MT	No	No	Endoscopy	No	No
Imaizumi, et al. [26]	No	Xeno (human) iPSC FISH	No	H&E vG MT	panCK AFP ALPP	Collagen and elastin density Retention of hydrogel	No	No	No
Morisaki et al. [61]	FACS (CD29, CD90, CD45)	Allo AD-MSC GFP tracing	HAS1 HAS2 HAS3 COL1A1 COL3A1 MMP1 MMP8 FGF2 HGF VEGFa	vG AB	No	Collagen and HA density	No	No	No
Bartlett et al. [54]	No	Xeno (human) BM-MSC Human β-actin expression	COL1A2 COL3A1 FN1 FMOD LPL OCN HAS HYAL2 IL1β IL17β, TGF-β1, TNF INγ αSMA	MT VvG AB	No	Relative collagen, elastin and HA content	No	No	No
Goel et al. [48]	No	Allo AD-MSC FISH, PCR (Y CS) TUNEL labeling for apoptotic cells	No	H&E MT	No	No	No	No	No
Svistushkin et al. [62]	No	Auto BM-MSC ND	No	H&E PR O	Collagen type I and III	LP thickness Thickness of collagen fibrils Scored analysis of 11 morphological parameters Collagen type I/III ratio	No	No	No
Hertegård et al. [22]	– FACS (CD73, CD90, CD105, HLA-I, CD14, CD34, CD45, HLA-II) – Migration and invasion assay – qPCR (HAS, HYAL, MMP) – ELISA (IL8, HGF, IL6, VEGF, TGF-β1)	Xeno (human) MSC FISH	No	AB	Collagen type I	Fibrosis, inflammation, MPS intensity Scored fibrosis analysis	No	No	No

*AB* Alcian blue, *AD-MSC* adipose tissue-derived mesenchymal stromal cells, *AFP* alpha 1 fetoprotein, *AG* adipogenic, *Allo* allogeneic, *ALPP* placental alkaline phosphatase, *Auto* autological, *BM-MSC* bone marrow-derived mesenchymal stromal cells, *CG* chondrogenic, *COL1A1* procollagen I type, *COL1A2* procollagen alpha 2, *COL3A1* procollagen III type, *CS* chromosome, *dAD-MSC* differentiated adipose tissue-derived mesenchymal stromal cells, *EM* epiglottis mucosa, *EP* epithelium, *ESC* embryonic stem cells, *FGF2* fibroblast growth factor 2, *FN1* fibronectin (MA), *FMOD* fibromodulin, *FN* fibronectin, *FSP-1* fibroblast specific protein 1, *GAG* glycosaminoglycans, *HA* hyaluronic acid, *HAS1* hyaluronan synthase-I, *HAS2* hyaluronan synthase-II, *HAS3* hyaluronan synthase-III, *HGF* hepatocyte growth factor, *HLA* human leucocyte antigen, *HS* histological staining, *HYAL3* hyaluronidase 3, *FACS* fluorescence-activated cell sorter analysis, *ICC* immunocytochemical staining, *IHC* immunohistochemical staining, *IL1β* interleukin 1β, *IL17β* interleukin 17β, *INγ* interferon γ, *iPSC* induced pluripotent stem cells, *LP* lamina propria, *LPL* lipoprotein lipase, *LV* lentivirus, *MA* molecular analysis, *MMP1* matrix metalloproteinase 1, *MMP8* matrix metalloproteinase 8, *MPS* mucopolysaccharides, *MT* Masson trichrome, *MTT* 3-(4,5-dimethylthiazol-2-yl)-2,5-diphenyltetrazolium bromide, *O* orcein, *OC* osteocalcin, *OG* osteogenic, *panCK* pancytokeratin, *PR* picrosirius red, *RO* reticulin and orcein, *SEM* scanning electron microscopy, *SMA* smooth muscle actin, *TGF-β1* transforming growth factor-beta 1, *TNF* tumor necrosis factor, *VEGFa* vascular endothelial growth factor type A, *vG* van Gieson, *VvG* Verhoeff-Van Gieson, *Xeno* xenogeneic

More extensive in vitro studies are conducted in case of utilizing cells after differentiation and within the composition of hydrogel systems or tissue engineered constructs. In particular, in the study by Hu et al., the morphology and expression of vimentin and fibronectin are compared for VF fibroblasts and fibroblast-like cells differentiated from the adipose tissue-derived MSC [22]. A number of studies estimate the stem cells’ ability to differentiate in the adipogenic, chondrogenic, and osteogenic direction, as well as their clonogenic potential [27, 41, 46].

To visualize cells and cell-based structures, the use of fluorescent microscopy is described with preliminary staining of the cell nuclei with 4′6-diamidino-2-phenylindole (DAPI) and immunofluorescent labeling for marker proteins (vimentin, pancytokeratin, etc.), as well as the use of electron scanning microscopy [46, 47]. Before the implantation into the VF, researchers, in particular, Shiba et al., relatively often perform a comparative histological analysis of the formed equivalents and the intact VF mucosa using different staining protocols [47]. Besides, Hertegård et. al. conducted an extensive analysis of the influence of a hyaluronic acid-based hydrogel on MSC in the presence of different anti-inflammatory cytokines (IL1b, IL8, or CCL4). The authors estimated such parameters as the rate of migration and invasion, the level of expression of hyaluronidase (HYAL), hyaluronan synthase (HAS), and matrix metalloproteinases (MMPs), as well as the level of IL6, IL8, HGF, TGFb1, and VEGF in the secretome, using the immunocytochemical method, qPCR, and ELISA [22].

## Assessment of outcomes after the implantation of cell-based products

### Detection of the implanted cells

Detection of the implanted cells is one of the most important stages in the estimation of the experimental results and is performed in the majority of relevant studies. The cells’ destiny after implantation is of interest from the viewpoint not only of their survival, but also of the mechanism of their action. For instance, the hypothesis about an antifibrotic effect generated due to paracrine and autocrine mechanisms acquires growing popularity currently [28, 63, 64].

The choice of the visualization technique depends mostly on the cell type and source. In a number of studies, cells of genetically modified animals are administered, namely, of mice expressing green fluorescent protein (GFP). Hence, these cells may be relatively easily detected by fluorescent microscopy when analyzing histological preparations [40, 49, 52, 60, 61]. Besides, cells may be labeled with fluorochrome trackers (e.g., Cell Tracker CM-DiI), or using genetic modification via transfection with the corresponding plasmids or a lentiviral vector transduction so that they express fluorochrome proteins, in particular, GFP and RFP [27, 38, 41, 45]. In the case of human cells, the method of fluorescent in situ hybridization (FISH) is frequently applied which uses labeled human DNA [22, 23, 26]. This technique along with PCR may detect male cells through the Y-chromosome DNA after their injection into the female VF, this concept was demonstrated in the study by Goel et al. [48]. Such a cross-sex experimental design with the use of allogeneic cells allows one to avoid the restrictions of the traditional cell visualization methods related to the reduction of the dye concentration due to cell division and unstable expression of fluorochrome proteins.

However, cell counting and statistical processing of such data are performed in a relatively small number of studies, which may be due to their absence at the control time which is usually 4–12 weeks from the moment of implantation [22, 25, 40]. In this connection, the techniques of in vivo real-time cell tracking are of particular interest. For example, Choi et al. detected cell fluorescence in vivo to reveal the implanted cells [25]. Besides the direct cell visualization, indirect methods of cell detection have been described. In particular, in the study by Bartlett et al. the absence of long persistence of human bone marrow-derived MSC implanted in a rabbit’s VF was evaluated by the level of human β-actin expression.

The additionally evaluated parameters, besides the cell survival, are proliferation, apoptosis, expression of proteins, etc. For example, Johnson et al. estimated cell proliferation by the Ki-67 mitotic marker, and the presence of apoptosis by staining with FITC-labeled 20-deoxyuridine 50-triphosphate (dUTP) and expression of smooth muscle actin (SMA) [52]. Peng et al. confirmed differentiation of VF-implanted MSC derived from the epiglottis mucosa into fibroblasts and myofibroblasts using vimentin and SMA expression [27].

### Molecular analysis

Molecular biology techniques of analysis, in particular PCR, open great opportunities for the extensive studies on the mechanisms of the VF repair after injecting cells and cell-based structures. In the experimental VF restoration, they are used to achieve two goals: analysis of the expression of genes coding extracellular matrix proteins (collagens type I and II, fibronectin) and related enzymes (hyaluronic acid synthase, MMP-1 and 8) [52, 60, 61], and establishing the expression level of growth factors and other chemokines modulating the VF regeneration (TGF- β1, FGF2, HGF, VEGF, IL1β, IL17β, TNF, etc.) [60].

The comparative analysis of the published results is difficult due to different cell types and their sources, carriers, observation times and other specifics of experimental design. However, statistically significant results in different combinations are most frequently found for collagens, fibronectin and TGF-β1 [40, 52, 54, 60, 61].

Such a technique as PCR allows avoiding the consumption of a large amount of a material and determines a wide spectrum of indices with their possible statistical processing. Nevertheless, the obtained results may be used only indirectly, since the current data are not sufficient to reliably confirm the correlation between the level of expressed proteins and the final functional result of the VF restoration.

### Morphological and immunohistochemical analysis

The morphological analysis represents one of the basic methods revealing the tissue structure specifics and proving the efficiency of the VF repair. Its protocols in regard to the estimation of the VF regeneration in general coincide with those for other approaches, although there are certain peculiarities.

Based on the analysis of publications, one may distinguish the following three directions of the histology studies: (1) a “classical” histology analysis with a set of different stains, taking into account the specifics of the VF mucosa’s ECM; (2) a histochemical analysis of the ECM components; and (3) a comparative analysis of quantified qualitative characteristics.

The most widespread stains applied in the histology analysis are hematoxylin–eosin and Masson trichrome for the general tissue structure, picrosirius red for revealing collagen, orcein and van Gieson stain to visualize elastic fibers, and alcian blue to identify glycosaminoglycans [25–27, 38, 41, 46, 47, 50, 53, 54, 60–62]. When applying immunohistochemistry staining, antibodies to collagen type I and fibronectin are mainly used [27, 38, 41, 57]; in some studies, collagen type III, elastin, hyaluronic acid and decorin were also visualized [39, 62]. Application of these approaches reflects the known data on the structure of the native and scarred VF mucosa [4, 13, 20, 65]. Besides, to establish possible human iPSC differentiation into epitheliocytes during the damaged VF tissue invasion and rule out development of a tumor from undifferentiated cells, Imaizumi et al. utilized antibodies to keratin proteins and alpha 1 fetoprotein and placental alkaline phosphatase, respectively [26].

The morphometry approaches to improve the analysis objectivity are worth a separate discussion. The comparison of a relative amount of the ECM components is applied most frequently. To do this, the digitalized image of a histological section with a certain color due to the corresponding staining is processed to measure its area. Such an approach is illustrative in the experiments on the larynx and has provided statistically significant differences when estimating the results of the VF repair in many studies. First, this concept was applied to compare the general content of collagen, glycosaminoglycans and elastic fibers in the histology studies [27, 46, 50, 54, 60], and to compare the content of collagen type I and fibronectin in the immunohistochemistry studies [22, 38, 41, 57]. A semiquantitative score of the morphological signs with the use of subjective scaling is rather frequently utilized. Both the general level of fibrous changes in the tissues using 3- and 4-point scores and the degree of distinction of individual morphological criteria are assessed [22, 53, 57, 62]. Besides, another widely used parameter is the thickness of the VF mucosa’s lamina propria. In spite of the relative simplicity and statistically significant differences in one and the same study, the comparison of different studies shows that, in a number of cases, the conclusions appear contradictory. For example, Svensson et al., Valerie et al. and De Bonnecaze et al. observed thickening of the scarred mucosa in respect to the intact one and the values becoming close after the administration of cells [41, 53, 57]. In contrast, Hiwatashi et al. and Ohno et al. noted thickening of the VF mucosa with injected adipose tissue-derived and bone marrow-derived MSC as compared to the control (in the latter case, the finding may be explained by the use of a collagen sponge as a carrier) [40, 50].

The further extrapolation of the conclusions of experimental studies is also ambiguous due to the differences in the created pathology both in the form of hypertrophic scars and in the form of VF atrophy. An interesting approach to show statistically significant differences was presented in the study by Choi et al., in which the authors determined a scar index as a ratio of the content of red thickened collagen fibers to that of thinner green fibers in a polarized light microscope [25]. De Bonnecaze et al. suggested measuring the optical density of elastic fibers (fibers/5000 μm^2^) and calculating accumulated immunocompetent cells (inflammatory foci/mm^3^) as indices reflecting inflammatory infiltration and having statistically significant differences [41]. Besides, Morisaki et al. performed calculations of the density of hyaluronic acid and collagen at different sites along a VF that is of importance due to its proven anisotropic properties [61]. In our previous study, we suggested determination of a collagen type I to collagen type III ratio [62].

### Physical properties

The VF physical properties are directly related to the voice quality: the increased rigidity of tissues and alteration of their shape due to scarring lead to the violation of the generation of mucosal waves and VF vibration. The pool of the applied techniques may be arbitrarily divided to those measuring the mechanical or vibrational VF properties. It should be noted that both groups are rather scarcely presented in the experimental studies on the cell material administration to the VF.

The dominating technique of the first group is parallel plate rheometry providing such parameters as dynamic viscosity, (Pa s) and elastic modulus, (Pa) [23, 38, 57, 60]. Besides, De Bonnecaze et al. estimated the mechanical properties of tissues using an electrodynamic shaker via the parameter of the first natural frequency at the location closest to the VF tissue center. However, in general the VF mechanical properties may be determined with a wider set of techniques, including linear rheometry and extension tests, which is discussed in detail in a review by Miri [66].

Parallel plate rheometry allows one to obtain significant statistical differences with the mentioned parameters. However, the analysis is performed at the macro-level without a possibility of precisely measuring the VF mucosa’s lamina propria’s characteristics and the ECM proteins’ structure, although a number of studies mention an essential direct contribution of the collagen fibrils’ microarchitecture to the mechanical properties of the VF tissues. Indeed, the effect of the implanted cell material on the processes of collagen formation, tissue remodeling, and hence, the VF mechanical properties remain yet understudied [65, 67–69].

For the deep understanding of the morphology and mechanical properties of the intact and scarred VF mucosa’s lamina propria, scanning probe techniques such as atomic force microscopy are used [68–72]. In particular, we applied this technique to analyze the packing and thickness of VF collagen fibrils and their mechanical properties after administering autologous bone marrow-derived MSC to rabbits and found a statistically significant difference in the relative Young’s moduli between the control and experimental groups [62]. However, to obtain the absolute values of the mechanical parameters, it is reasonable to use indentation methods (AFM or nanoindentation) for a native sample under fluid.

The VF vibrational characteristics were analyzed only in a few studies [25, 38, 47, 50]. For the measurements, a high-frequency recording of the VF vibration in a larynx microsample was performed with the subsequent evaluation of the mucosal wave amplitude; the VF vibrational activity was induced by passing an air flow through the trachea. The lowered mucosal vibration amplitude of the damaged VF, as a rule, became higher and closer to the normal values, after the cell product administration, as shown by Kim et al., Choi et al. and Ohno et al. [25, 38, 50].

Along with the VF vibration amplitude, a frequency spectrum of vibrations is also an extremely important parameter. In the clinical practice, acoustic voice analysis has acquired a wide use as a method of phonation objectivization [44, 73, 74]. Nevertheless, there exist techniques for the direct examination of the VF vibrations, which become of great importance in the animal experiments due to acoustic analysis being non-applicable [75–79]. In particular, in the study by Luizard et al., laser vibrometry was applied for the analysis of an artificial model VF’s vibrations, and the obtained results were presented [80].

### Clinical assessment

The analysis of the larynx visualization data after surgical interventions is of major importance for adequate extrapolation of the experimental results to clinical studies. Such parameters as tissue edema, severity of reactive phenomena, and regeneration dynamics directly correlate with the safety and a positive course of the rehabilitation period. Together with the functional results, they define the potential advantages of the regenerative medicine technology for patients. Nevertheless, the majority of studies did not include such as analysis, or it was presented as a short conclusion to the photograph of the final control point [25, 38, 60]. In only very few studies, dynamic endophotographs of a larynx are displayed, for example, in the study by Peng et al. [27]. It would also be interesting to perform a qualitative investigation by the basic macroscopic signs applied in the clinical practice. Such as approach was described in the study by Lee et al., in which, along with the extensive description of the injured VF healing dynamics, the authors note the formation of granulomas, VF atrophy, heterogeneity of the surface and scarring [45].

## Translation into clinical practice

As of now, first clinical trials have been conducted based on some experimental studies. In particular, the results of a pilot study on the treatment of the VF scars using autologous BM-MSC have been published [33]. The study included 16 patients with uni- and bilateral VF scars of various etiologies, including those after a surgical treatment of the laryngeal cancer (in 5 patients, the glottal closure defect during phonation reached 1.5–2 mm). In the conditions of microlaryngoscopy, 0.5–2 million MSC in a saline or in a hyaluronic acid gel were implanted into the VF defect following the scar resection. No adverse effects were noted during the first year after the surgery; an essential improvement of the VF vibrations was observed in 62–75% of patients, according to the data of high-speed laryngoscopy and the phonation pressure threshold; the VHI (voice handicap index) was significantly improved in 8 patients, while the other patients did not experience noticeable changes [33]. Besides, in the study by Mattei et al. (2020), 1 mL of the adipose tissue-derived stromal vascular fraction were administered to 6 patients with the VF scars and 2 patients with the VF sulcus in the area of the scar defect (no excision of the scar tissue was performed). In 12 months, the VHI was improved in all the patients, and in 7 patients this improvement was 18 and more points [34]. A number of questions were raised also in the commentary of this research group to the pilot clinical study by Hertegård [33], including questions regarding the necessity of the scar excision prior to the MSC administration and inclusion of patients after cordectomy due to the VF cancer. In response, Hertegård et al. refer to the positive results of their own experimental studies, in which the scar resection was performed, and point to the high safety profile of MSC which justified the possibility of treating oncological patients. At the same time, the authors emphasize that the results were inferior in the case of extensive defects. Both research groups note that further studies in this direction are needed [81, 82].

## Prospects

Based on the performed analysis, we can distinguish a number of directions which may be of interest for further studies.

The first direction regards the alternative ways of cell delivery, in particular, by transthyroidal injection, which is widely used in administering fillers for the VF medialization in unilateral VF paralyses [83, 84]. Such an approach provides an opportunity for several injections which may be instrumental in experimental studies of the dose-dependent effects. When potentially used for cell therapy in the clinical practice, it does not require laryngoscopy, general anesthesia and hospital stay of patients.

Besides, an important direction is the optimization of the experimental design, which includes application of various dosages and repetitions of cell injections in the framework of one and the same study. Indeed, the study quality may be essentially improved when a single unified experimental model and identical surgical steps are used by a single research group, thus decreasing the number of non-systematic errors. Currently, it is impossible to compare the efficiency of different doses of a cell product in the VF restoration.

Not only the choice of the cells’ type (including their sources) is of interest for the further studies, but also the choice of their form for implantation (cell suspension or self-organizing cell structures). In our opinion, cell spheroids are a promising form, representing 3D spheroidal self-organizing cell structures. In a number of studies on the repair of injuries in other tissues, they have shown high efficacy and facilitated the regeneration at the defect site [85–88]. Besides, they have a pronounced angiogenic potential and sustain the cells’ phenotype [89–92].

It is noteworthy that the assessment of the results should include the analysis of macroscopic changes and intraoperative hemorrhage, which directly affect the cells’ viability at the implantation site and the therapy results. The results of such an analysis allow selecting the optimal carriers with high adhesion to the wound surface and hemostatic properties. Application of noninvasive techniques, indirectly characterizing the microcirculatory bed status, such as photoplethysmography, is also of interest [93].

## Conclusions

As of now, preclinical studies remain in demand and an important element of the development of new ways for the treatment of any lesions, including those of the VF. However, the requirements imposed upon their design are rather hazy and dictated by the country. For example, in the European Union countries they are collected in the EMA regulation guide, in the USA—in the guide of the “Preclinical Assessment of Investigational Cellular and Gene Therapy Products” by FDA [94, 95].

Nevertheless, based on the results of our literature review, we have noted an increase in the relative fraction of in vitro studies in the published data, which are dedicated to the characterization of the used cell material. It is probably related to the fact that the main trend in the development of new strategies for the VF restoration consists in application of various carriers which provide enhanced cell survival and local modulation of regeneration processes [25, 26, 96]. The application of new biomaterials as carriers requires extensive studies using adequate animal models.

Among the approaches to the assessment of the VF restoration efficiency, histological and immunohistochemical staining to reveal the basic ECM components (collagen, elastin and glycosaminoglycans) remain the main techniques, with the subsequent morphometry analysis. At the same time, scant studies include the attempts to find the mechanisms of the repair processes, and only few studies describe the morphological alterations of the larynx at the macroscopic level, although it is of great importance for the clinical practice. Besides, in one study only the attention was paid to the potential effect of the administered cells on organs and tissues of other body systems to which they may have tropism [54].

As the mechanical and vibrational indices of VF, most authors use the Young’s modulus of a VF macrospecimen and the amplitude of the mucosal wave generated by artificially induced vibration of a larynx sample. In doing so, almost no researchers study the mechanical properties directly in the scar region.

Thus, many questions remain currently unanswered regarding both the mechanisms of the VF regeneration upon the administration of cells, selection of the most efficient cell source, selection of a carrier and the approaches to the design of in vivo studies and the choice of the most adequate model. The development of unified recommendations on the conduction of preclinical studies and adequate estimation of the results taking into account the VF morphofunctional specifics is necessary for the effective translation of the obtained results to the clinical practice.

## Data Availability

Not applicable.
